# Functional Disability in Psychiatric Patients With Deliberate Self-Harm as Compared to a Clinical Control Group

**DOI:** 10.3389/fpsyt.2021.579987

**Published:** 2021-04-06

**Authors:** Magnus Nilsson, Lars-Gunnar Lundh, Åsa Westrin, Sofie Westling

**Affiliations:** ^1^Department of Clinical Sciences Lund, Psychiatry, Lund University, Clinical Psychiatric Research Center, Region Skåne, Lund, Sweden; ^2^Department of Psychology, Lund University, Lund, Sweden

**Keywords:** non-suicidal self-injury, deliberate self-harm, functional disability, quality of life, daily functioning

## Abstract

**Background:** Deliberate self-harm (DSH) is a common behavior in psychiatric populations. However, little is known regarding how DSH impacts daily life. The concept of functional disability, adopted by the World Health Organization (WHO), refers to the impact of disorders on six domains of daily functioning. The aim of the current study was to explore the functional disability of psychiatric patients with DSH as compared to a psychiatric control group.

**Methods:** 32 psychiatric patients with DSH and 31 psychiatric patients without DSH were assessed with regards to demographic information, functional disability, psychiatric illness, DSH, general cognitive functioning, and measures of psychopathology. Group comparisons were made by means of *t*-tests, Mann-Whitney-tests, and Chi-square tests. Correlation analyses were done to assess the association between measures of psychopathology and functional disability.

**Results:** The results indicated that patients with DSH had a lower ability to self-care as compared to the patients without DSH (*p* = 0.001, *d* = 0.90). Also, the patients with DSH reported a significantly higher number of days when they were totally unable to carry out usual activities in the past month (*p* = 0.008, *d* = 0.70) and that they were admitted in an inpatient setting significantly more days over the past year compared to the patients without DSH (*p* < 0.001, *d* = 0.58). The group with DSH was significantly younger (*t* = 3.00, *p* = 0.004) and reported significantly more BPD-symptoms (*p* = 0.013, *d* = 0.64) as well as higher current suicidality (*p* < 0.001, *d* = 1.32) compared to the group without DSH. The group with DSH also included a significantly higher number of patients diagnosed with borderline personality disorder (χ2 = 13.72, *p* < 0.001). There were no differences between the groups regarding general cognitive functioning or severity of depression. More research is needed to understand the underlying factors involved.

## Introduction

Contemporary psychiatry is predominantly focused on assessing and describing symptoms, categorizing them into diagnoses which serve as understandable constructs for clinicians and patients (1). These constructs, in turn, lie as a foundation for clinicians to direct treatments and interventions. Beyond the diagnostic constructs defined by the DSM-V structure, the National Institute for Mental Health (NIMH) has proposed a framework for research: the Research Domain Criteria (RDoc). This framework integrates knowledge from multiple research sources and suggests that research should focus on specific trans-diagnostic phenomena rather than diagnoses (2). In line with this approach, as well as of clinical interest, the current study focuses on the phenomenon of Deliberate self-harm (DSH) rather than the diagnoses that usually are associated with it. DSH is a common trans-diagnostic behavior in psychiatry (3–5) and is a predictor for future suicide attempts (6). The behavior is associated with borderline personality disorder (BPD) where it is one of the core criteria (1). A plethora of research has improved our knowledge about DSH and its mechanisms providing directions for future treatment research on concepts, such as self-compassion, stimuli aversion, and self-care (7).

In order to understand the impact of mental health challenges, more information is required. Individuals with psychiatric disorders may experience difficulties adapting and organizing their lives beyond their psychiatric symptoms (8). Further, symptom remission does not always entail an improvement of functioning (9, 10). Yet, daily functioning is rarely studied as an outcome measure (11). The current paper explores the relationship between self-harm and daily functioning.

The concept of functional disability refers to the impact of disorders on daily functioning. The World Health Organization (WHO) has developed a system for health based on function called the International Classification of Functioning, Disability and Health (ICF). Within the *activity and participation* dimension six domains have been listed: *cognition* (understanding and communication), *mobility* (ability to move and get around), *self-care* (ability to attend to personal hygiene, dressing and eating, and to live alone), *getting along* (ability to interact with other people), *life activities* (ability to carry out regular responsibilities at home, work, and school), and *participation in society* (ability to engage in community, civil and recreational activities). These domains are meant to cover most daily activities and be applicable to any health condition in various cultures (12). ICF has been operationalized through the WHO Disability Assessment Schedule (WHODAS 2.0), an instrument assessing disability within each domain.

Previous research related to DSH has focused on functional disability in BPD. These studies have indicated that functional impairment seems to be greater in BPD in comparison with other personality disorders (13). The functional disability in BPD tends to improve slightly over the long term, but these improvements are smaller for women (14). There are fewer studies using comparison groups with clinical conditions as well as gender matching (13, 14) which limits the conclusions that can be drawn regarding the functional disability in individuals with BPD.

To our knowledge, no published research has explored the impact of self-harm on functional disability. Likewise, no other published record has included the concept and domains of functional disability adopted by the WHO (12) for this population. Considering the high prevalence of DSH in psychiatric populations there is a need to gain insight into its impact on functional disability using a comprehensive, well-established, and validated method. Thus, the current study aims to explore the functional disability in psychiatric patients with DSH compared to a control group of psychiatric patients without DSH. Since deficits in daily functioning could be the effect of other aspects of psychopathology than DSH, the current study will also explore the association between functional disability and measures of psychopathology.

## Materials and Methods

Ethical approval was provided by the regional ethical review board at Lund University (Reg. No. 2014/626). Convenience samples of 32 participants with current psychiatric disorders with DSH (DSH), and 31 participants with current psychiatric disorders but without current or past DSH (non-DSH; NDSH) were recruited. The recruitment of the DSH-group was done consecutively through a specialized team for the treatment of DSH 2015–2016 and during certain time periods consecutively through the intake at a general psychiatric outpatient clinic 2016–2019. Participants in the DSH-group were recruited first. Since all participants with DSH were registered as women at birth, only women were recruited in the NDSH-group. The recruitment of the NDSH-group was done during certain periods consecutively through the intake at a general psychiatric outpatient clinic 2016–2019 and consecutively through a psychiatric research unit 2018–2019. Additionally, information about the study was sent out by email to clinicians from 2014 to 2019 reminding them about the possibility to inform individuals about participation. Thus, participants were included both through the intake and through their clinicians.

In the current study we used the term DSH suggested by Hawton, Rodham, Evans, & Weatheralls (15) who define self-harm as all acts with non-fatal outcome in which an individual deliberately did one or more of the following:

- Initiated behavior (for example self-cutting, jumping from a height) with which they intended to cause self-harm;
- Ingested a substance in excess of the prescribed or generally recognized therapeutic dose;
- Ingested a recreational or illicit drug that was an act that the person regarded as self-harm;
- Ingested a non-ingestible substance or object.

To be included in the DSH group the individual had to have self-harmed on at least three occasions over the past 6 months. To be included in the NDSH group the individual should have self-harmed on at most two occasions, and not within the past 6 months.

Due to the established significant impairment in daily functioning associated with bipolar I disorder and schizophrenia (16), participants with these disorders were excluded. The same applied for individuals with substance use or alcohol addiction disorder within the past 12 months (17) as well as individuals with ADHD, ADD, and autism (18–21). To limit the impact of comorbid depression on functional disability, individuals with current severe depression, defined as a total Montgomery Asberg Depression Rating Scale (MADRS) score of >34, also were excluded. Finally, participants with a history of any non-psychiatric disorder that could affect the current daily functioning were excluded. As BPD is associated with negative effects on daily functioning (13), it can be argued that this group should also have been excluded. However, since DSH is closely associated with BPD we decided that studying DSH without including BPD would exclude a significant and important subgroup in this population.

All participants were assessed by the same licensed psychologist (Author “MN”) trained to administer each of the measures. A psychiatrist (Author “SW”) was available for queries regarding diagnosis. All participants who completed the research procedure were reimbursed with 50€.

## Measures

### Demographics

All participants were asked to report age, vocational and relationship status, level of education, and current cohabitants.

### Diagnostic Measures and Measures of Psychopathology

The MINI International Neuropsychiatric Interview 6.0 (MINI 6.0) (22) and the Structured Clinical Interview of the DSM-IV 2 (SCID-2) (23) were used to determine diagnoses. Diagnostic measures for DSM-5 were not available in Swedish when the first participant was enrolled and diagnosed. Thus, the outcome of the MINI 6.0 was subsequently adapted to the DSM-5. In MINI 6.0, section B consists of questions regarding suicidal ideation and behavior over the past month as well as previous suicidal behavior. The total score on this section was used as a measure of suicidality (24, 25). The Borderline Symptoms List-23, BSL-23 (26) is a scale divided into three sections. The first section, which contains 23 items regarding typical borderline symptoms, produces a total score between 0 and 92. This section was used to measure borderline symptoms. The following two sections contain a supplementary rating scale and 11 items that measure related behavioral problems. Level of depression was measured using the Montgomery Asberg Depression Rating Scale, MADRS (27). This 10-item rating scale consists of items with a Likert scale ranging from 0 to 6. A total score between 34 and 60 indicates severe depression, 20–24 moderate depression and 7–19 mild depression.

### Measures of DSH

The Inventory of Statements about Self Injury, ISAS (28) is a self-report questionnaire assessing statements about self-injury. In the first section of ISAS the participant is asked to report life-time frequencies of thirteen methods of self-harm, including twelve pre-defined methods and an undefined item where the participant could describe any previous unlisted method. The following section include questions about age of onset, pain and impulsivity followed by 39 statements regarding the participant's reasons for self-harming. The participant is asked to rate the accuracy of these statements (0 = not relevant, 1 = somewhat relevant or 2 = very relevant). The statements are added to 13 composite category scores between 0 and 6 points: *affect regulation, anti-dissociation, anti-suicide, marking distress, self-punishment, autonomy, interpersonal boundaries, interpersonal influence, peer bonding, revenge, self-care, sensation seeking*, and *toughness*.

In addition, the participants were asked to rate any other self-harming behaviors, irrespective of suicidal intention: jumping from heights, intoxication through medication and swallowing sharp objects, as well as any other self-harming behavior that had not been assessed through previous questions. Thus, the total of life-time incidents was generated by combining the results from the first section of ISAS with the supplementary questions.

### Measures of Functional Disability

The 36-item self-administered World Health Organization Disability Assessment Schedule, WHODAS, 2.0 (29) was used to measure functional disability. The scale measures functioning in six domains: *Cognition, Mobility, Self-care, Getting along, Life activities*, and *Participation in society*. It also includes questions about the number of days in the last month when these difficulties have been present and how many of these days the respondents have been totally or partially unable to carry out their normal activities. Alongside WHODAS 2.0, the number of sick days and hospitalization days in the last year were obtained as measures of functional disability.

### Measures of General Cognitive Functioning

For an estimation of general cognitive functioning, five subtests from the Wechsler Adult Intelligence Scale 4, WAIS-IV, (30) were used: Digit Span, Block Design, Vocabulary, Information and Symbol Coding. The composite standard scores of these subtests were used to estimate the general cognitive functional level.

### Statistical Analysis

In order to determine whether the results were normally distributed, skewness and kurtosis were calculated for all variables. Chi-square tests were used to assess differences in education, employment status and relationship status. Independent sample *t*-tests were used to assess group differences in sick days, level of borderline symptoms, level of depression, suicidality, functional disability, and general cognitive functioning. A Mann-Whitney test was used to assess the difference in number of hospitalization days. Cohen's *d* was used to measure the effect size for the significant results. Spearman's rho was used to analyze the correlation between measures of functional disability and measures of psychopathology. To correct for depression and borderline symptoms on significant outcome measures, multiple regression analyses were done using a dummy variable (DSH present or not present) and MADRS and BSL-23 as separate covariates.

## Results

### Tests of Normality

The results for skewness and kurtosis were within an acceptable range, below two and seven, respectively (31) except for the number of hospitalization days in the past year.

### Demographics

Chi square tests revealed no significant group differences in relationship or vocational status nor for cohabitants or education level (Table 1). The DSH-group was significantly younger (M = 24.31, SD = 5.42) than the NDSH-group (M = 29.23, SD = 7.43) (*t* = 3.00, *p* = 0.004).

**Table 1:** Demographic characteristics for the two study groups.

	**DSH**	**NDSH**
*n*	32	31
Age, *M* (*SD*)	24.3 (5.4)	29.2 (7.4)^*^
**Education**		
Grade School	8	3
High School	18	19
University	6	9
**Vocational status**		
Employed or studying	23	21
Unemployed	9	10
**Relationship status**		
Never been in a relationship	7	6
Not currently but been in the past	12	9
Current relationship less than 1 year	5	2
Current relationship more than 1 year	8	14
**Cohabitants**		
Single person household	9	13
Lives with spouse or common-law	5	8
Lives with parents	10	5
Lives with adults other than parents	8	5

* *p < 0.05. M, Mean; SD, Standard deviation; DSH, study group with deliberate self-harm; NDSH, study group without deliberate self-harm*.

### Group Comparisons for Psychopathology, Functional Disability, and General Cognitive Functioning

Table 2 illustrates the results on measures of psychopathology, functional disability, and general cognitive functioning level (Table 2).

**Table 2:** Distribution of diagnoses and results for measures of psychopathology, functioning measures and general cognitive functioning level.

	**DSH**	**NDSH**	***t/Z/ χ2***	***p***	***d***
*n*	32	31			
MADRS, M (SD)	18.4 (7.2)	17.9 (5.4)	*t =* 0.32	0.754	
BSL-23, M (SD)	44.6 (20.8)	31.5 (20.0)	*t =* 2.53	0.013^*^	0.64
Suicidality, MINI 6.0 M (SD)	14.9 (11.5)	3.7 (3.4)	*t =* 5.19	<0.001^***^	1.32
Sick days past year, M (SD)	134.4 (149.9)	71.7 (124.1)	*t =* 1.52	0.134	
Hospitalized days past year M (SD) (SD)	18.9 (41.2)	1.7 (9.0)	Z = 4.13	<0.001^***^	0.58
**WHODAS 2.0, M (SD)**					
Cognition	7.4 (3.8)	8.1 (5.1)	*t =* 0.64	0.526	
Mobility	4.4 (3.7)	5.3 (3.5)	*t =* 0.95	0.348	
Self-care	4.9 (2.9)	2.7 (1.9)	*t =* 3.54	0.001^***^	0.90
Getting along	7.2 (3.5)	7.8 (4.5)	*t =* 0.61	0.544	
Life activities	17.9 (8.4)	13.5 (6.6)	*t =* 1.80	0.076	
Participation in society	14.3 (5.6)	12.6 (5.2)	*t =* 1.23	0.225	
Days of difficulties past month	23.9 (7.4)	20.7 (8.0)	*t =* 1.63	0.108	
Days totally unable past month	10.8 (10.1)	4.9 (6.5)	*t =* 2.75	0.008^**^	0.70
Days partially unable past month	11.9 (8.7)	12.7 (9.5)	*t =* 0.38	0.706	
WAIS IV-5 subtest, M (SD)	98.9 (8.6)	103.1 (19.2)	*t =* 1.76	0.083	
**Primary psychiatric diagnosis**					
Borderline personality disorder	17	3	χ2 = 13.72	<0.001^***^	
Bipolar II disorder	5	9	χ2 = 0.48	0.489	
Major depressive	7	6	χ2 = 0.006	0.895	
Obsessive compulsive disorder	3	4	χ2 = 0.20	0.656	
Posttraumatic stress disorder	0	2	χ2 = 2.13	0.144	
Generalized anxiety disorder	0	2	χ2 = 2.13	0.144	
Social phobia	0	2	χ2 = 2.13	0.144	
Panic disorder	0	1	χ2 = 1.05	0.306	
Body dismorphic disorder	0	2	χ2 = 2.13	0.144	

* p < 0.05,

** p < 0.01,

*** p < 0.001.

*M, Mean; SD, Standard deviation; DSH, study group with deliberate self-harm; NDSH, study group without deliberate self-harm; WAIS, Wechsler Adult Intelligence Scale; MADRS, Montgomery Asberg Depression Scale; BSL-23, Borderline Symptom List-23; WHODAS, World Health Organization disability assessment schedule; MINI, Mini International Neuropsychiatric Interview*.

There were significantly more participants with BPD in the DSH-group (χ^2^ = 13.72, *p* < 0.001). All participants in the clinical groups fulfilled the criteria for one or more psychiatric diagnoses. Participants in the DSH-group were diagnosed with: recurring major depressive disorder (*n* = 18), bipolar II disorder (*n* = 8), panic disorder (*n* = 11 of which 5 had concurrent agoraphobia), social phobia (*n* = 4), obsessive-compulsive disorder (*n* = 3), posttraumatic stress disorder (*n* = 8), bulimia (*n* = 3), avoidant personality disorder (*n* = 2), dependent personality disorder (*n* = 3), obsessive-compulsive personality disorder (*n* = 3), paranoid personality disorder (*n* = 2), and borderline personality disorder (*n* = 17). In the NDSH-group the following diagnoses occurred: recurring major depressive disorder (*n* = 18), bipolar II disorder (*n* = 8), current panic disorder (*n* = 4 of which 1 had concurrent agoraphobia), agoraphobia without panic disorder (*n* = 1), social phobia (*n* = 4), obsessive-compulsive disorder (*n* = 4), posttraumatic stress disorder (*n* = 3), bulimia (*n* = 2), avoidant personality disorder (*n* = 1), and borderline personality disorder (*n* = 3). No differences in levels of depression were found between the groups. The DSH-group reported significantly higher scores on the BSL than the NDSH group with a moderate effect size (*p* = 0.013, *d* = 0.64) and had a higher level of suicidality according to the MINI with a strong effect size (*p* < 0.001, *d* = 1.32).

The number of sick days during the past year ranged from 0 to 365 in both the DSH-and the NDSH-group. The number of hospitalization days ranged from 0 to 210 in the DSH-group and 0–50 in the NDSH-group. The DSH-group reported being hospitalized significantly more days than the NDSH-group in the last year with a moderate effect size (*p* < 0.001, *d* = 0.58). Further, the DSH-group reported significantly more difficulties in *self-care* the past month than the NDSH-group with a large effect size (*p* = 0.001, *d* = 0.90) as well as a significantly higher number of days in the last month being totally unable to carry out usual activities (*days totally unable)* with a moderate effect size (*p* = 0.008, *d* = 0.70). There were no significant differences between the groups in the other domains as measured by WHODAS 2.0 or in estimated cognitive functioning level between the groups.

### Measures of DSH

The mean number of lifetime DSH-incidents was 866 (SD = 1,501) with a range from 13 to 7,781. To interfere with wound healing/e.g., picking scabs was the most commonly occurring behavior followed by cutting and grazing. Highest score for reasons for DSH was *affect regulation* (M = 5.2, SD = 1.2) followed *self-punishment* (M = 4.0, SD = 1.9) and *marking distress* (M = 2.8, SD = 1.9). This is in line with previous research om research for self-harming behavior (32, 33).

### The Relationship Between Measures of Psychopathology and Measures of Functional Disability

*Days totally unable* correlated significantly with borderline symptoms (R_s_ = 0.47, *p* < 0.001), level of depression (R_s_ = 0.40, *p* = 0.001), and suicidality (R_s_ = 0.32, *p* = 0.008). *Self-care* from WHODAS 2.0 correlated significantly with borderline symptoms (R_s_ = 0.44, *p* < 0.001), level of depression (R_s_ = 0.32, *p* = 0.010), and suicidality (R_s_ = 0.47, *p* < 0.001).

The logistic regression analyses showed that DSH predicted low *self-care* independently of depression (β = 0.40, *p* = 0.001) and independently of borderline symptoms (β = −0.31, *p* = 0.01). DSH also predicted *days totally unable* independently of depression (β = 0.32, *p* = 0.005), although when controlled for borderline symptoms the effect was no longer significant (β = 0.21, *p* = 0.076). In addition, however, as seen in Table 3, depression and borderline symptoms were independent predictors both of *self-care* and *days totally unable*. (Table 3).

**Table 3:** Regression analysis: The impact of depression, borderline symptoms and self-harm on self-care and days totally unable.

	**Unstandardized coefficients**		**Standardized coefficients**					
**Model**	***B***	***SE***	**β**	***p***	***R^**2**^***	***R^**2**^*adjusted**	***F***	***p***
MADRS+DSH on *self-care*					0.29	0.27	12.74	<0.001^***^
MADRS	2.10	0.57	0.40	0.001^**^				
DSH	0.15	0.05	0.35	0.002^**^				
BSL-23+DSH on *self-care*					0.28	0.25	11.44	<0.001^***^
BSL-23	0.04	0.01	0.34	0.004^**^				
DSH	1.61	0.61	0.31	0.01^**^				
MADRS+DSH on *days totally unable*					0.28	0.26	11.83	<0.001^***^
MADRS	0.59	0.16	0.42	<0.001^***^				
DSH	5.61	1.94	0.32	0.005^**^				
BSL-23+DSH on *days totally unable*					0.24	0.22	9.60	<0.001^***^
BSL-23	0.16	0.05	0.38	0.002^**^				
DSH	3.80	2.10	0.21	0.076				

*N = 63*.

** p < 0.01,

*** p < 0.001.

*DSH, presence of self-harm; MADRS, Montgomery Asberg Depression Scale; BSL-23, Borderline Symptom List-23; SE, Standard Error*.

## Discussion

The current study investigated whether psychiatric patients with DSH have different levels of functioning in daily life than psychiatric patients without DSH. The results indicate that, after controlling for depression and borderline symptoms, the patients in the DSH-group experience significantly more functional disability, specifically regarding *self-care*. The questions in this domain cover difficulties getting dressed, eating, washing themselves as well as spending a few days by themselves. Furthermore, the DSH-group reported a significantly greater number of days the past month when they were totally unable to carry out their normal activities. This applied when correcting for depression but not for borderline symptoms. Further, the DSH-group spent more days admitted to a hospital as well as higher suicidality, as compared to the NDSH-group. The results offer insight into the daily challenges of individuals with DSH and might be used to adapt interventions for self-harm.

The test groups did not differ significantly in general cognitive functioning, education or employment. There was no significant difference in the level of depression between the DSH- and the NDSH-group. The results also remained significant after correcting for depression which taken together suggests that the difference in functional disability results was not due to depressive symptoms.

The results raise questions of other explanations as to why differences in functional disability occurs. One possible explanation might be that the DSH-group had significantly more borderline symptoms than the NDSH-group. Both *self-care* and *days totally unable* from WHODAS 2.0 correlated significantly with borderline symptoms. Thus, the increased functional disability in the DSH-group could be due to the higher level of borderline symptomatology. The effects of BPD on functional disability could speak in favor of such an interpretation (11, 13, 14). What speaks against such an explanation, however, is that the group difference in *self-care* remained significant after correcting for borderline symptoms whereas, *days totally unable* was close to but not significant.

Another possible explanation is that the DSH-group had a higher level of suicidality as measured by MINI 6.0. Suicidality was significantly related to *self-care* and *days totally unable*. The relation between suicidal behavior and functional disability has previously been established in older adults (34). There are several possible reasons for these associations. One possibility is that there is an underlying factor explaining both higher suicidality and functional disability. Alternatively, the relationship between suicidality and functional disability is reciprocal, where high suicidality affects the functional disability and vice versa.

A third explanation may be that the differences in functional disability are due to other factors, such as the role of stigma, social rejection, and self-beliefs. DSH has been associated with stigma (35, 36) and mental health stigma may lead to social rejection (37) as well as social withdrawal in various clinical groups (38). This could also be the case for persons with DSH. The DSH-group in the current study however, did not differ from the NDSH-group with regards to *Getting along* and *Participation in society*. This indicates that they did not experience more difficulties with regards to social inclusion as compared to the NDSH-group. Thus, self-harm specifically did not appear to decrease the reported social inclusion beyond the stigma that is associated with a mental health condition. However, DSH has been associated with negative attitudes toward oneself (7) and negative self-attitudes and self-stigmatization are also associated with a decrease in self-efficacy (39). This in turn could lead to less *self-care* and less motivation for goal-directed behaviors (37). Thus, it is possible that the self-stigma associated with DSH could contribute to greater withdrawal and lower *self-care* in individuals with DSH as compared to others.

A fourth possibility is that the lack of *self-care* could hypothetically fulfill self-punishing purposes similar to what many individuals report as the motivation behind DSH (7). In the current study, self-punishment was the second most commonly reported reason to self-harm which could points toward such an interpretation.

Individuals with BPD tend to present more severe symptoms in self-report forms as compared to interview-based ratings (40). Since BPD was more frequent in the DSH-group, a fifth possibility that might partly explain the results, is that this group reported subjectively more functional disability than the NDSH-group as the result of some kind of negative bias. The lack of significant difference in *days totally unable* when correcting for BPD-symptoms could speak in favor of this possibility.

## Limitations

This study of how functional disability is related to DSH specifically includes a number of challenges. Firstly, to separate DSH from the many other symptoms is difficult. A matching in terms of psychiatric diagnoses would be preferable so that both psychiatric groups would show a similar distribution of psychiatric diagnoses. This is difficult to achieve for various reasons. The association between DSH and BPD is close. Most patients with BPD tend to engage in DSH (41) so it is extremely difficult to study DSH in psychiatric patients in isolation from other aspects of BPD. In the current study, the two groups had different levels of borderline symptoms and there were differences with regards to their psychiatric diagnoses. Still, they had similar levels of education, depression, and vocational status. Further, we believe that the comparison between the DSH- and the NDSH-group is justified since the participants in both groups had severe and multiple diagnoses. In Swedish settings, specialized psychiatry primarily focuses on treatment resistant or otherwise moderate to severe cases of mental illnesses. Although there was a difference in borderline symptoms, the difference in s*elf-care* between the groups remained significant even after correcting for these symptoms. Thus, despite the limitations, there is support to claim that the self-harm, specifically, may play a role for some aspects of functional disability. To fully explore the effects of DSH on functional disability by differentiating DSH from BPD would require a bigger sample and different design.

A general limitation is that persons with ADHD, ADD, autism, schizophrenia, substance use disorders and bipolar I disorder were excluded from the study groups. BPD, which was more prevalent in the DSH-group, is associated with significantly more comorbidity (42). The implications of this could be that the study groups potentially represent a relatively healthy sample in comparison with other more severely mentally ill individuals. We deemed it necessary however, to limit the number of factors that otherwise could affect the functional disability. Considering the prevalence of comorbid diagnoses, high suicidality and frequent hospitalization days, we believe that the study samples do represent the content of specialized psychiatry in a fairly accurate way.

A limitation to the conclusion of this study is the difference in age between the DSH- and the NDSH-group. This difference in age might explain the difference in functional disability, as younger people with psychiatric disorders have potentially had less time to adapt to, cope with, and receive help for their mental illness. However, for this study, we consider this unlikely since a recent related meta-analysis on this topic for persons with BPD only has indicated small such improvements with age, especially for women (14).

With limitations taken in consideration the results indicate experientially different functional disability for individuals with DSH as compared to individuals without DSH in a psychiatric sample. More research is needed to explore the underlying factors contributing to the dysfunction. This could provide valuable insights needed as functional disability might need environmental adaptations that are not a part of standard treatment for DSH. Beyond these research endeavors, the results could provide valuable input and understanding to clinicians working with this population as well as for the individuals with experience of DSH.

## Data Availability Statement

The datasets presented in this article are not readily available because of Swedish confidentiality laws. Requests to access the datasets should be directed to the authors.

## Ethics Statement

This study was approved by and approved by Regional ethical review board at Lund University (Reg. No. 81 2014/626). The patients/participants provided their written informed consent to participate in this study.

## Author Contributions

MN designed the protocol, recruited participants, collected data, analyzed the results, drafted, and substantively revised the manuscript. SW made substantial contributions to the design, recruitment process, and substantively revised the manuscript. L-GL made substantial contributions to the design and statistical analysis, interpreted the results, and substantively revised the manuscript. ÅW interpreted the data and substantively revised the manuscript. All authors approved the submitted version and agreed both to be personally accountable for their own contributions and ensued that questions related to the accuracy or integrity of any part of the work, even ones in which the author was not personally involved, were appropriately investigated, resolved, and the resolution documented in the literature.

## Conflict of Interest

The authors declare that the research was conducted in the absence of any commercial or financial relationships that could be construed as a potential conflict of interest.
